# Ethnopharmacological study on *Adenosma buchneroides* Bonati inhibiting inflammation via the regulation of TLR4/MyD88/NF-κB signaling pathway

**DOI:** 10.1007/s13659-024-00458-8

**Published:** 2024-06-04

**Authors:** Yuru Shi, Xiaoqian Zhang, Shengji Pei, Yuhua Wang

**Affiliations:** Department of Economic Plants and Biotechnology, Yunnan Key Laboratory for Wild Plant Resources, Kunming Institute of Botany, Chinese Academy of Sciences, Lanhei Road 132, Heilongtan, Kunming, 650201 Yunnan China

**Keywords:** Fleagrass, Anti-inflammatory activities, TLR4/MyD88/NF-κB signaling pathway, Ethnopharmacology

## Abstract

*Adenosma buchneroides* Bonati, also known as fleagrass, is an important medicinal plant used by the Akha (Hani) people of China for treating inflammation-related skin swelling, acne, and diarrhoea, among other conditions. In this study, we aimed to evaluate the anti-inflammatory activities and explore the molecular mechanisms of fleagrass on treating skin swelling and acne. The results demonstrated that fleagrass inhibited the enzymatic activities of 5-LOX and COX-2 in vitro, and decreased the release of NO, IL-6, TNF-α, and IL-10 in the LPS-induced RAW264.7 macrophages. The levels of proteins associated with the nuclear factor-kappa B (NF-κB) pathway were examined by western blotting and immunofluorescence, demonstrating that fleagrass downregulated the expression of TLR4, MyD88, NF-κB/p65, and iNOS and blocked the nuclear translocation of NF-κB/p65. Furthermore, fleagrass exhibited acute anti-inflammatory activity in paw oedema models. The results confirm that fleagrass exhibits remarkable anti-inflammatory activity and can be used in alleviating inflammation, suggesting that fleagrass has the potential to be a novel anti-inflammatory agent.

**Graphical Abstract:**

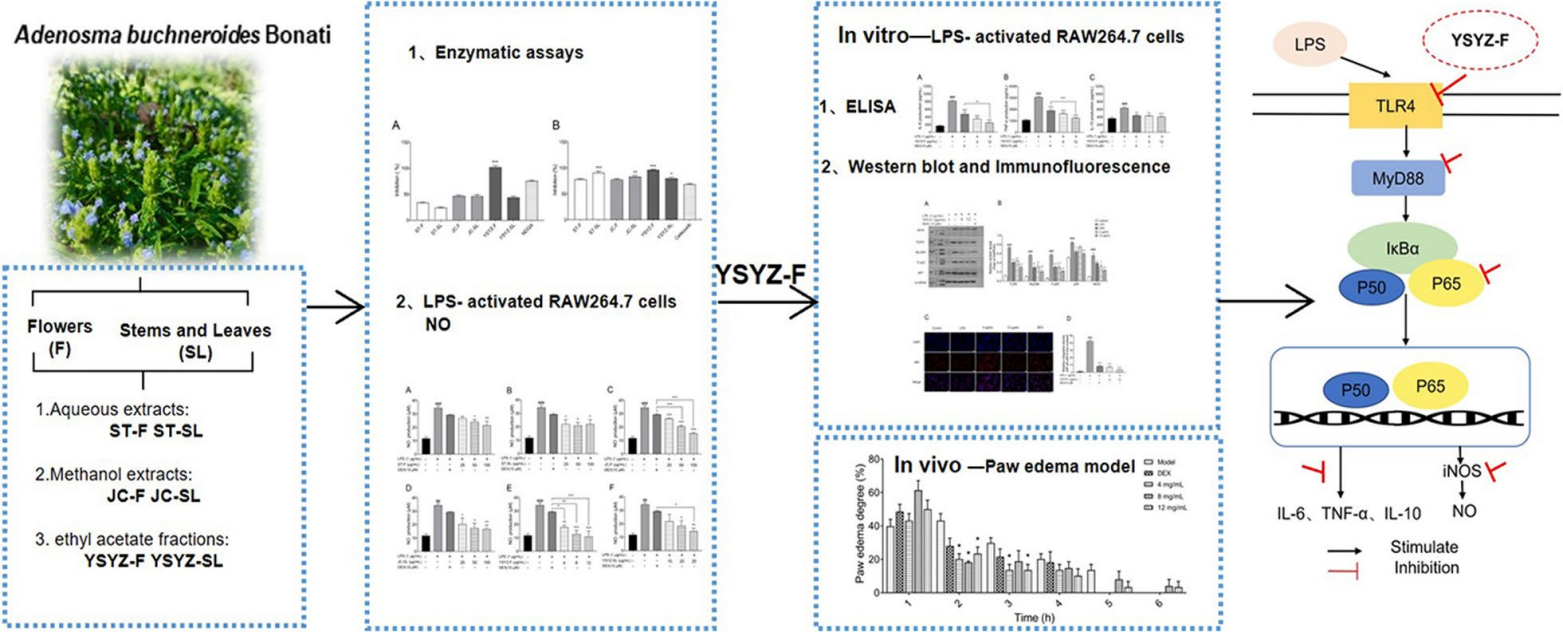

## Introduction

*Adenosma buchneroides* Bonati is an aromatic annual herb with a strong fragrance [1]. In 1980, Chinese pioneer ethnobotanist Prof. Shengji Pei first discovered this unique plant in Akha (Hani) villages during his ethnobotanical investigation in Xishuangbanna, Yunnan, China. Its vernacular name is ‘Lao-wo-suo-du’ among Akha villagers in production area. *A. buchneroides* is a valuable plant for Akha people who are fond of its scent and magic role of insects repel effect. Akha people wear this plant as a piece of ear jewellery or on caps as decoration and wear its scent as a perfume; using this plant is a cultural identity symbol of being an Akha tribal individual. The Akha people put the bundles of the plant in their rooms or spread them on beds to ward off fleas or other insects; thus, Prof. Pei named it fleagrass [2]. Furthermore, it is also a vital medicinal plant that helps Akha people to maintain their family’s health. From 2016 to 2018, we re-conducted a comprehensive systematic ethnobotanical survey of fleagrass in Xishuangbanna Dai Autonomous Prefecture of China and Phongsaly Province of the Lao PDR. In addition to the three previously documented uses, we documented the applications of fleagrass as a ritual plant and a condiment [3]. Moreover, the fleagrass was found to be processed in diverse ways by Akha people to make medicine for treating inflammation-related diseases, including skin conditions such as itching, swelling, and acne, as well as influenza and diarrhoea. Concretely, Akha people crush fresh fleagrass and apply it topically to soothe itching and swelling caused by insect bites; they also make fleagrass potions to scrub insect bite wounds and acne off the skin [3].

To understand the traditional use of fleagrass as an insecticide by the Akha people, we studied its chemical composition and mosquito-repellent activity. We identified 26 chemical components in the essential oil of fleagrass using gas chromatography (GC) and GC–mass spectrometry [4]. The main components respectively were *γ*-terpinene (34.86%), carvacrol (22.2%), *p*-cymene (12.1%), carvacrol methyl ether (11.87%), and *β*-Bisabolene (7.96%). Subsequently, three mosquito-repellent active components, namely carvacrol, carvacrol methyl ether, and adenosmin A, were isolated from the essential oil using a bioactivity-guided separation strategy. The essential oil and three components were tested for adult *Aedes albopictus* repellency by performing an in-cage mosquito repellent bioassay. The results showed that, compared with the positive control N, N-diethyl-meta-toluamide (MED = 0.031 ± 0.014 mg/cm^2^), the essential oil (MED = 0.019 ± 0.007 mg/cm^2^) and carvacrol (MED = 0.011 ± 0.000 mg/cm^2^) exhibited stronger mosquito repellent activities. Furthermore, adenosmin A (MED = 0.063 ± 0.027 mg/cm^2^) and carvacrol methyl ether (MED = 0.125 ± 0.054 mg/cm^2^) exhibited mosquito-repellent activity [4]. After nearly 10 years of in-depth research, the mosquito-repellent activity and chemical basis of fleagrass have been unravelled, and relevant products, such as creams and sprays, have been developed. These study findings were successfully translated into practical applications in 2022. However, to the best of our knowledge, the traditional medicinal uses of fleagrass showed it as a potent anti-inflammatory agent, which has not been scientifically verified.

Inflammation is a defensive response of the immune system of human body that aims to eliminate harmful stimuli, including microbial infections, chemical substances, and tissue damage while facilitating tissue repair [5, 6]. Excessive and persistent inflammatory responses are closely associated with the occurrence and development of various diseases including cancer, rheumatoid arthritis, diabetes, cardiovascular diseases, and neurodegenerative diseases [7–11]. Non-steroidal anti-inflammatory drugs (NSAIDs), immunosuppressive drugs, and corticosteroids are conventional therapeutic agents used for treating inflammatory diseases [12]. However, their long-term use may lead to many side effects, including alimentary tract haemorrhage, anaphylaxis, and liver and kidney function damage [13–16]. Therefore, developing effective anti-inflammatory drugs with fewer side effects is essential. Compounds from natural resources are reliable raw materials for new pharmaceuticals [17], and traditional medicines that have been validated based on their traditional use for centuries hold tremendous potential as research subjects.

Thus, herein, we aim to evaluate the anti-inflammatory activities of fleagrass in vitro and in vivo. Furthermore, the underlying molecular mechanism will be studied in-depth using a lipopolysaccharide (LPS)-activated RAW 264.7 macrophage model of inflammation. Hopefully, validating the traditional use of fleagrass in relieving skin inflammation can provide a theoretical basis for isolating bioactive compounds and developing new anti-inflammatory drugs.

## Results

### The extracts inhibited the enzymatic activity of 5-LOX and COX-2

**Fig. 1:**
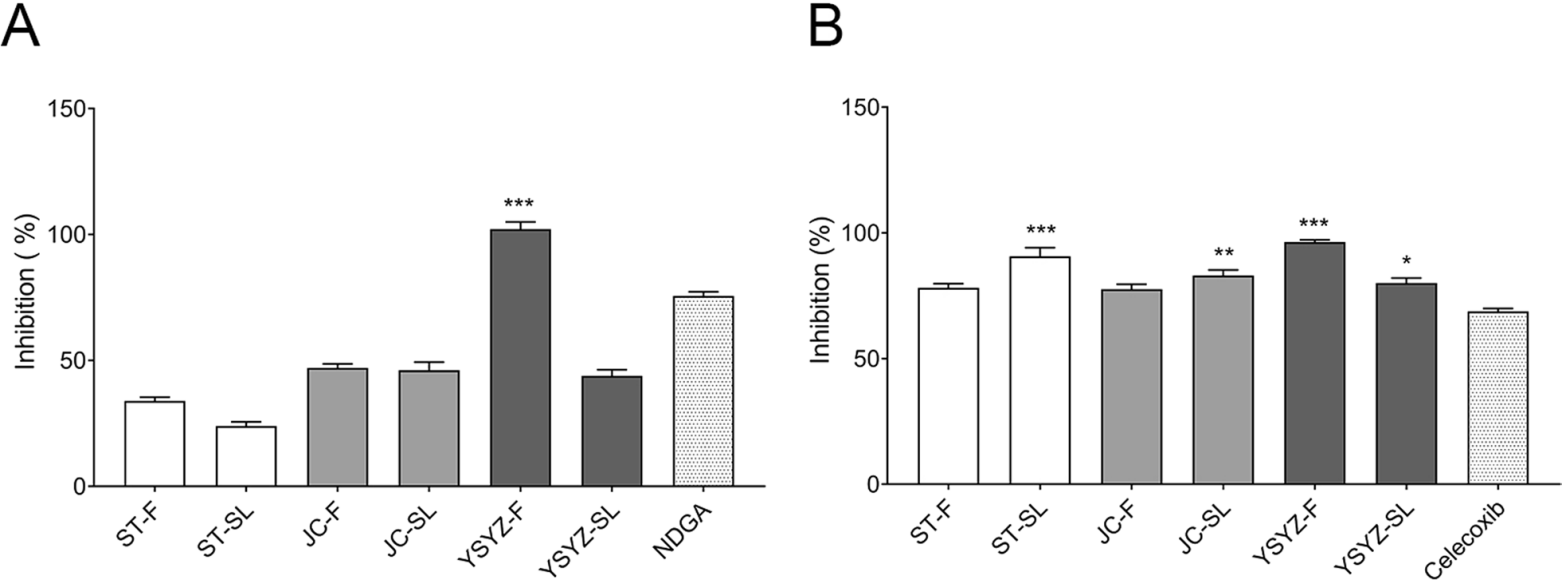
Enzymatic inhibition activity of fleagrass extracts on 5-LOX and COX-2. Fleagrass extracts inhibited the enzymatic activity of 5-LOX (**A**) and COX-2 (**B**). Data are represented as mean ± SEM (n = 3). *p < 0.05, **p < 0.01, and ***p < 0.001 vs. positive control group

The six extracts exerted inhibitory effects on the enzymatic activity of 5-LOX at a concentration of 250 μg/mL (Fig. 1A). The inhibition rates of ST-F (33.9%) and ST-SL (23.94%) on the enzymatic activity of 5-LOX were between 20 and 40% and those of JC-F (47.07%), JC-SL (46.07%), and YSYZ-SL (43.87%) were between 40 and 50%. Specifically, YSYZ-F exerted a more significant inhibitory effect on the enzymatic activity of 5-LOX with the highest inhibition rate of 102.15%, whereas NDGA showed an inhibition rate of 75.60% at a concentration of 75 μM. All six extracts exerted significant inhibitory effects on the enzymatic activity of COX-2 at a concentration of 50 μg/mL (Fig. 1B), and these inhibition rates were greater than 70%. The inhibition rate of celecoxib was 68.76% at a concentration of 10 μM. These results showed that all six extracts exerted good inhibitory effects on the enzymatic activity of 5-LOX and/or COX-2 in vitro. Among all the tested extracts, YSYZ-F exhibited potent inhibitory effects on the enzymatic activities of 5-LOX and COX-2 with inhibition rates greater than 95%.

### Effects of the extracts on cell viability

**Fig. 2:**
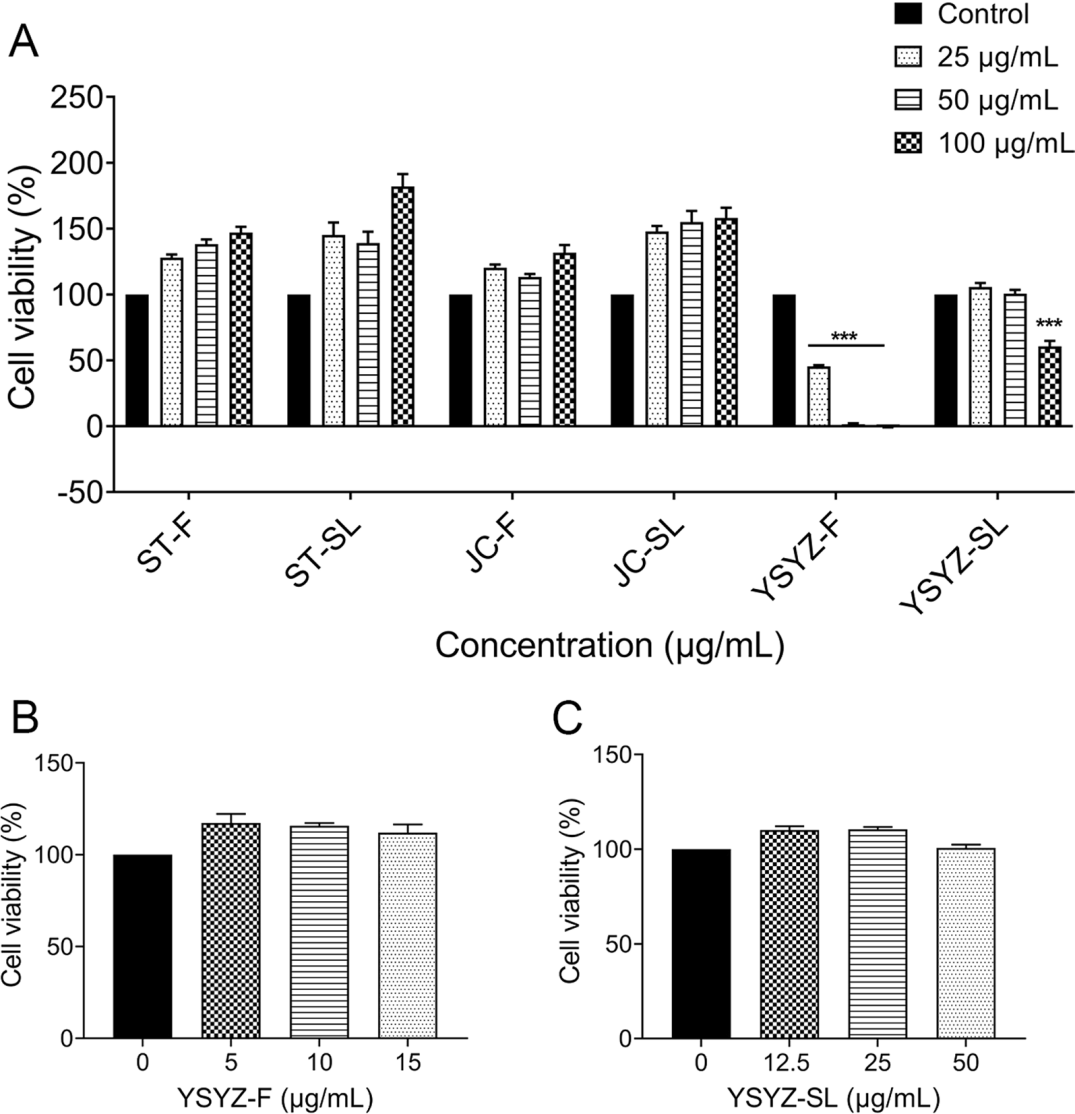
Effects of different extracts of fleagrass on cell viability. **A** Quantitative analysis of cell viability of 6 extracts (25, 50 and 100 μg/mL). **B** Quantitative analysis of cell viability of YSYZ-F (5, 10 and 15 μg/mL). **C** Quantitative analysis of cell viability of YSYZ-SL (12.5, 25 and 50 μg/mL). Data are represented as mean ± SEM (n = 3). ***p < 0.001 vs. control group

RAW 264.7 macrophages were respectively treated with different concentrations of ST-F, ST-SL, JC-F, JC-S, YSYZ-F, and YSYZ-SL along with LPS (1 μg/mL) for 24 h, and their viability was determined by performing the CCK-8 assay. Within a concentration range of 100 μg/mL and lower, ST-F, ST-SL, JC-F, and JC-SL exerted no significant inhibitory effect on cell viability (Fig. 2A). However, YSYZ-F and YSYZ-SL significantly reduced cell viability at concentrations of 25 μg/mL and 100 μg/mL, respectively. Thus, their concentrations were further decreased, and we found that YSYZ-F did not affect cell viability within a concentration range of 15 μg/mL and below (Fig. 2B), whereas YSYZ-SL had no effect within a concentration range of 50 μg/mL and below (Fig. 2C).

### The extracts decreased the level of NO in LPS-induced RAW264.7 macrophages

**Fig. 3:**
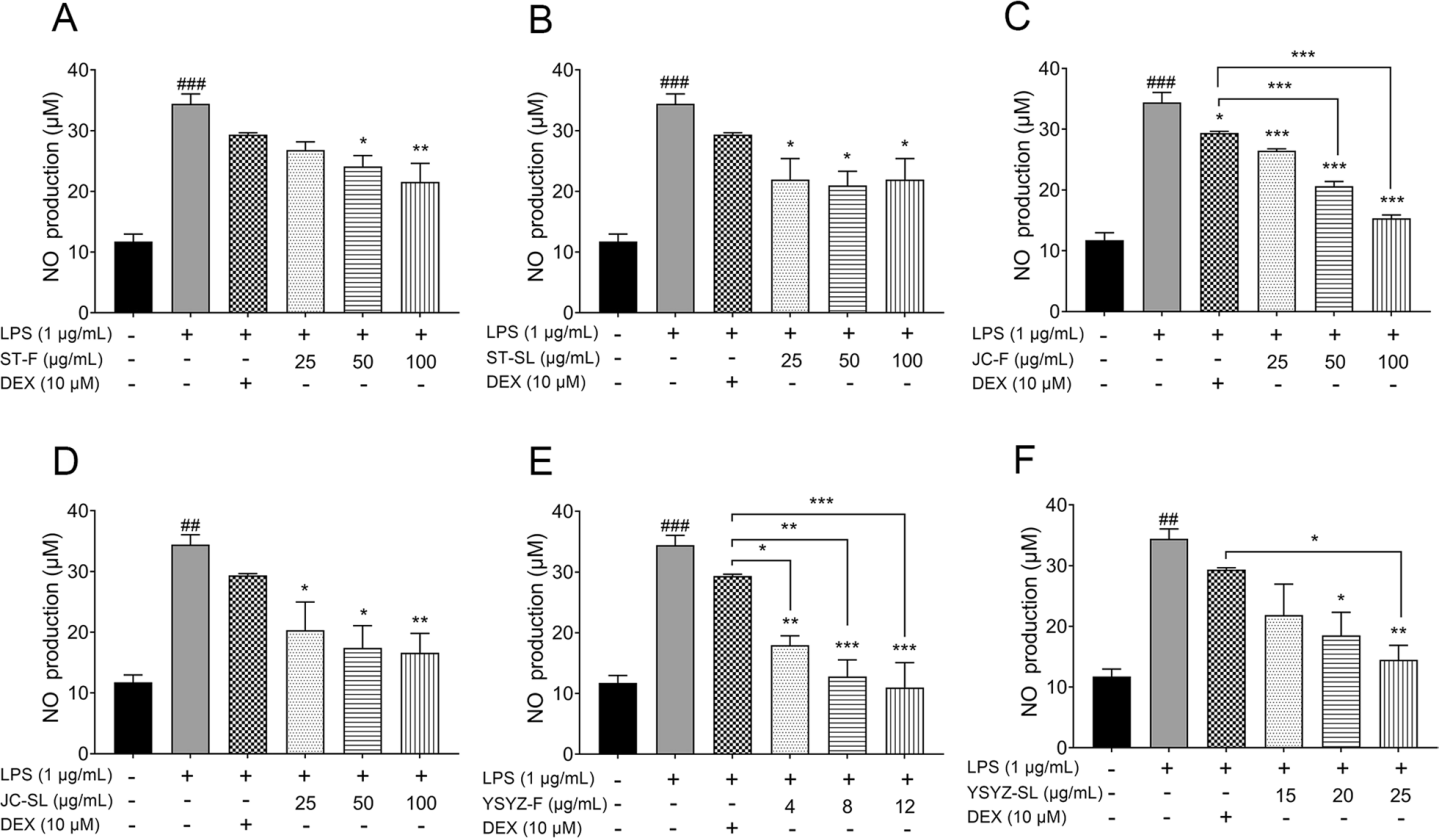
ST-F (**A**), ST-SL (**B**), JC-F (**C**), JC-SL (**D**), YSYZ-F (**E**), and YSYZ-SL (**F**) significantly inhibited LPS-induced NO production in RAW 264.7. Data are represented as mean ± SEM (n = 3). ^##^p < 0.01, ^###^p < 0.001 vs. control group; *p < 0.05, **p < 0.01, and ***p < 0.001 vs. LPS group

Based on the results of the cell viability assay, the concentrations of ST-F, ST-SL, JC-F, and JC-SL were both set to 25, 50, and 100 μg/mL, whereas the concentrations of YSYZ-F were set to 4, 8, and 12 μg/mL and those of YSYZ-SL were set to 15, 20, and 25 μg/mL. The level of NO in the supernatant was determined by the Griess assay. Compared with the control group, LPS (1 μg/mL) significantly increased NO secretion in the RAW264.7 macrophages, and ST-F, ST-SL, JC-F, JC-SL, YSYZ-F, and YSYZ-SL significantly inhibited NO production (Fig. 3). YSYZ-F exerted the most significant inhibitory effect at a concentration of 12 μg/mL, with the highest inhibition rate of 67%. The inhibition rates of ST-F, ST-SL, JC-F, and JC-SL at 100 μg/mL were 37%, 37%, 55%, and 51%, respectively and that of YSYZ-SL at 25 μg/mL was 58%. These results suggested that all six extracts exhibited a good inhibitory effect on NO production in the LPS-induced RAW 264.7 macrophages.

### YSYZ-F decreased the levels of IL-6, TNF-α, and IL-10 in the LPS-induced RAW264.7 macrophages

**Fig. 4:**
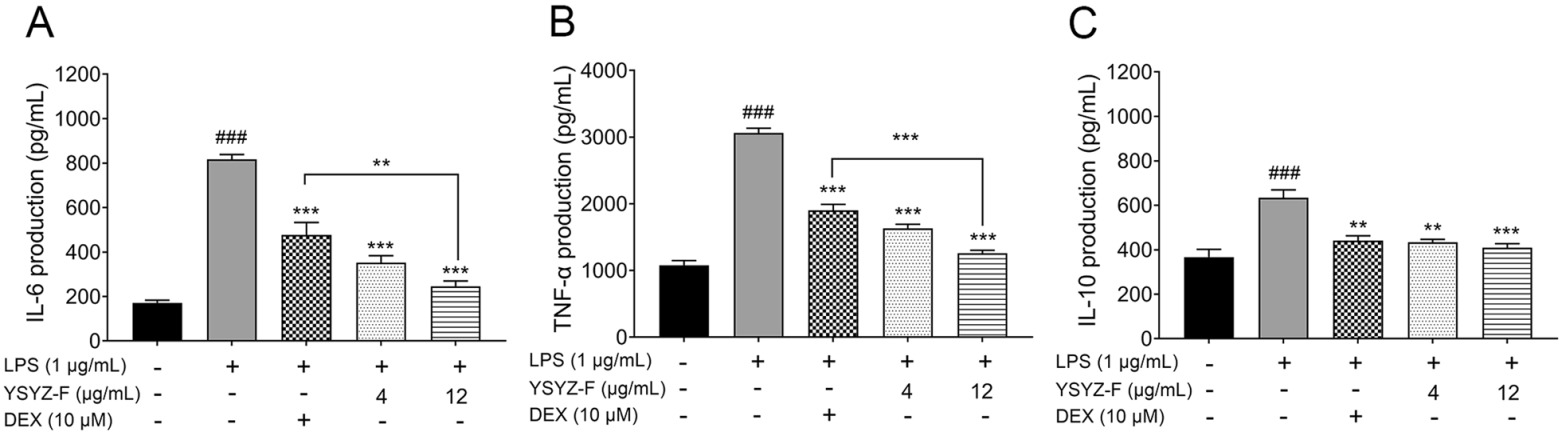
Effects of YSYZ-F on the levels of IL-6 (**A**), TNF-α (**B**) and IL-10 (**C**) in LPS-stimulated RAW264.7 macrophages. Data are represented as mean ± SEM (n = 3). ^###^p < 0.001 vs. control group; **p < 0.01, and ***p < 0.001 vs. LPS group

Because YSYZ-F significantly inhibited the enzymatic activities of 5-LOX and COX-2, as well, it significantly inhibited the production of NO in the LPS-induced RAW 264.7 macrophages, we further investigated its effects (4 and 12 μg/mL) on the production of IL-6, TNF-α, and IL-10 in the LPS-induced RAW 264.7 macrophages. LPS (1 μg/mL) significantly increased the secretion of IL-6, TNF-α, and IL-10, whereas YSYZ-F significantly inhibited the overproduction of these inflammatory factors in a dose-dependent manner (Fig. 4). YSYZ-F exerted more significant inhibitory effects on the production of IL-6 and TNF-α at a concentration of 12 μg/mL with the highest inhibition rates of 70% and 59%, respectively. DEX showed the inhibition rate of 42% and 37%, respectively, at a concentration of 10 μM. These results indicated that YSYZ-F exhibited potent anti-inflammatory activity in the LPS-stimulated RAW264.7 macrophages.

### YSYZ-F regulated inflammation via TLR4/MyD88/NF-κB signaling pathway

**Fig. 5:**
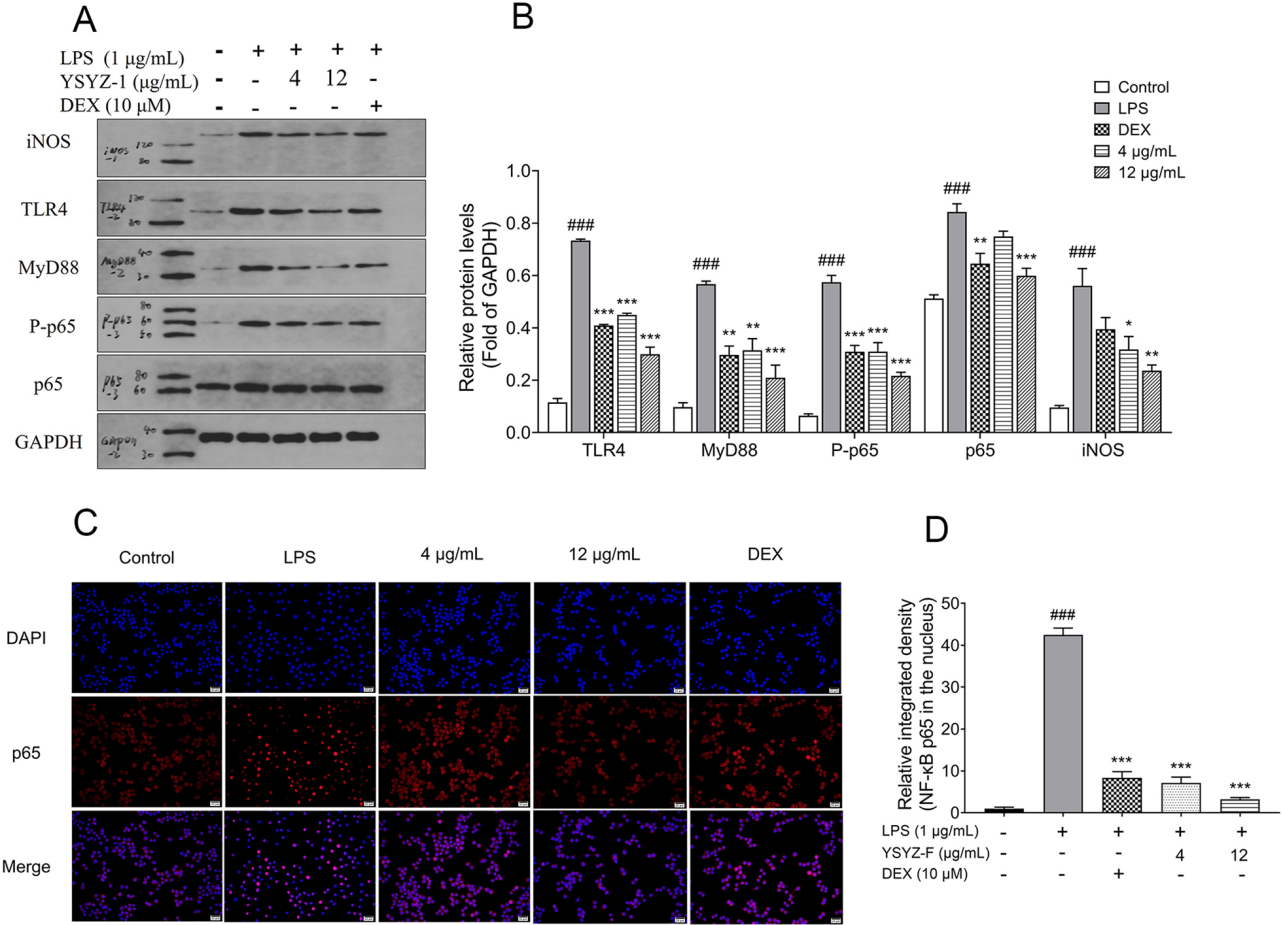
YSYZ-F inhibits TLR4/MyD88/NF-κB signaling pathway activation in LPS-induced RAW264.7 macrophages. **A** The relative protein expression was analyzed by western blotting. **B** Quantitative analysis of relative protein levels. **C** YSYZ-F inhibited the translocation of p65 to the nucleus in the LPS-stimulated RAW264.7 macrophages (×400). Scale bar: 20 μm. **D** Quantifications of the immunofluorescence pictures. Data are represented as mean ± SEM (n = 3). ^###^p < 0.001 vs. control group; *p < 0.05, **p < 0.01, and ***p < 0.001 vs. LPS group

To elucidate the molecular mechanism underlying inflammation regulation by YSYZ-F in LPS-stimulated RAW264.7 macrophages, we performed western blotting and determined the levels of related proteins. Compared with the control group, LPS stimulation increased TLR4, MyD88, NF-*κ*B p65, and NF-*κ*B P-p65 levels in the RAW264.7 macrophages (Fig. 5A, B). However, YSYZ-F remarkably decreased the levels of these proteins in a dose-dependent manner. Owing to its inhibitory effect on the production of NO, the effect of YSYZ-F on the level of iNOS, a downstream inflammatory protein, was investigated. The results showed that YSYZ-F (4 and 12 μg/mL) significantly decreased iNOS levels in a dose-dependent manner (Fig. 5A, B).

Subsequently, we investigated the effect of YSYZ-F on the nuclear translocation of the NF-κB p65 subunit protein using the immunofluorescence method. After LPS (1 μg/mL) stimulation, NF-κB p65 red fluorescence in the nucleus was significantly higher than that in the control group (Fig. 5C, D). However, YSYZ-F significantly decreased the intensity of NF-κB p65 red fluorescence in the nuclear region than that in the nuclei of LPS group in a dose-dependent manner (Fig. 5C, D). These results indicated that YSYZ-F significantly inhibited the translocation of NF-κB p65 to the nucleus in the LPS-stimulated RAW264.7 macrophages.

### In vivo anti-inflammatory activity of YSYZ-F

**Fig. 6:**
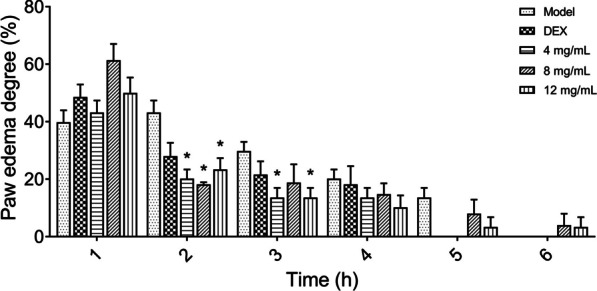
Topical anti-inflammatory effect of YSYZ-F on carrageenan-induced paw edema. Data are represented as mean ± SEM (n = 5). *p < 0.05 vs. model group

The in vivo anti-inflammatory activity of YSYZ-F was investigated using carrageenan-induced paw oedema models in mice. Carrageenan treatment significantly increased paw oedema and reached a maximum level in 2 h, with an oedema rate of 43.2% (Fig. 6). YSYZ-F (4, 8, and 12 mg/mL) significantly inhibited paw oedema in 2 h after carrageenan-induced oedema. The paw oedema rates of YSYZ-F at concentrations of 4, 8, and 12 mg/mL were 20.8%, 22.8%, and 23.4%, respectively, and the inhibitory effect was comparable to that of the positive control (DEX), which showed an oedema rate of 28.0%. These results suggested that YSYZ-F attenuated carrageenan-induced paw oedema in mice.

## Discussion and conclusion

AA (5, 8, 11, and 14-eicosatetraenoic acid) is an essential polyunsaturated fatty acid, and the human body primarily obtains it from the diet [18, 19], which is also the primary precursor in eicosanoid synthesis [6]. Eicosanoids, a type of lipid mediator, play a crucial role in initiating immunological responses, causing and alleviating inflammation [20, 21]. Because eicosanoids (leukotrienes and prostaglandins) are mainly produced from AA via COX and LOX pathways, 5-LOX and COX-2, two key enzymes in these pathways, are important targets for treating inflammation and related diseases [6, 20, 22]. Blocking or inhibiting the activities of these enzymes can reduce inflammatory biomarker levels, thereby alleviating inflammation. In the present study, the fleagrass extracts exerted good inhibitory effects on 5-LOX and COX-2 in vitro, indicating that fleagrass alleviated inflammation by suppressing the AA metabolic pathway. Medications that act on a single molecular target can result in undesired activity, toxicity, and drug resistance. Conversely, multitarget drugs possess the potential to avoid these issues while offering a better therapeutic profile [23]. Inhibiting either the 5-LOX or COX-2 pathway can potentially result in a metabolic switch to the other pathway and causing fatal side effects [24]. Dual COX-2/5-LOX inhibitors expand the protective range and avoid the serious side effects caused by traditional NSAIDs. Herein, among all test samples, YSYZ-F exerted the most significant inhibitory effect on both 5-LOX and COX-2, indicating its potential as a dual COX-2/5-LOX inhibitor. These findings provide a promising basis for developing novel anti-inflammatory drugs with enhanced safety profiles.

Macrophages play indispensable roles as immune defence cells in eliciting immune responses against allergies and inflammation [25]. LPS-induced RAW264.7 macrophage inflammation models are commonly used to evaluate the activity of anti-inflammatory drugs in vitro. LPS, an endotoxin, is a component of the outer membrane of gram-negative bacteria and induces pro-inflammatory responses and severe physiological reactions [26–28]. LPS interacts with TLR4 located on the plasma membrane of macrophages, initiating TLR4 dimerisation and subsequently recruiting MyD88 via the intracellular domain of TLR4, and ultimately activating various stress-sensitive pathways, such as NF-κB signalling [29–31]. NF-κB signalling plays a central role in inflammation and is involved in regulating diverse cytokines, cell adhesion molecules, and inducible enzymes such as NO, prostaglandin E2, TNF-α, IL-6, e-selectin, COX2, and iNOS [32, 33]. NF-κB primarily exists in the cytoplasm as a p50–p65 dimer and combines with inhibitory protein factor kappa B alpha (IκB-α). When RAW 264.7 cells are stimulated by LPS, IκB-α is phosphorylated and inactivated by IκB kinase, and subsequently, the activated p50–p65 dimer translocates into the nucleus, inducing the transcription of the corresponding target sequence and playing a role in secreting pro-inflammatory mediators [34–37]. The effective inhibition of the NF-κB pathway is one of the mechanisms by which numerous NSAIDs, such as aspirin, sulindac, tolfenamic, celecoxib, indomethacin, and ibuprofen, exert anti-inflammatory effects [38, 39]. The present findings showed that within a safe concentration range, the fleagrass extracts (ST-F, ST-SL, JC-F, JC-SL, YSYZ-F, and YSYZ-SL) exerted a significant inhibitory effect on NO production in the LPS-induced RAW264.7 macrophages. Particularly, YSYZ-F exhibited a significant dose-dependent inhibitory effect on IL-6 and TNF-α secretion, suggesting that fleagrass played an anti-inflammatory role by inhibiting the production of inflammatory mediators and cytokines. The level of IL-10, an anti-inflammatory cytokine, usually increases after drug treatment, which promotes the restoration of normal physiological conditions [40, 41]. However, the present results showed the opposite trend. Further research is required to verify and validate the present findings. The results of molecular mechanism investigation showed that YSYZ-F decreased the levels of TLR4, MyD88, NF-*κ*B p65, NF-*κ*B P-p65 and blocked NF-κB p65 nuclear translocation. These results demonstrate that fleagrass suppresses LPS-mediated inflammatory response in RAW264.7 macrophages partly due to inhibition of the NF-κB signaling. The occurrence and progression of inflammation are complex processes, and related drugs exert anti-inflammatory effects via multiple pathways. Future studies should explore other anti-inflammatory pathways and mechanisms of action of fleagrass. Furthermore, although the results showing that fleagrass alleviated paw oedema in mice, more in vivo inflammatory model are needed to verify the anti-inflammatory activity and mechanisms of fleagrass in the future.

Owing to the aromatic properties of fleagrass, its essential oils have attracted extensive attention from researchers. Shen et al. [2], Ma [4], and Xu et al. [42] isolated and identified various components of the essential oils, such as *γ*-terpinene, carvacrol, *p*-cymene, carvacrol methyl ether, and *β*-bisabolene. Most compounds exhibited excellent bioactivities such as insect-repellent, insecticidal, and antimicrobial properties, aligning perfectly with the traditional use of fleagrass by the Akha people to repel mosquitoes and insects. Xie [43] evaluated the anti-inflammatory activity of fleagrass essential oil using an LPS-induced RAW264.7 macrophage inflammation model, and the results showed that essential oil inhibited TNF-α release but could not inhibit IL-6 secretion. This experimental result is consistent with the present findings (data not shown). Compounds in the essential oil of fleagrass, which exhibited anti-inflammatory activity, were low in content, except for carvacrols, such as *α*-terpinene (1.77%), 4-terpineol (0.16%), and *β*-pinene (0.06%), and different studies reported varied carvacrol contents [2, 4, 42]. These results suggested that the essential oil of fleagrass may not be an important component for exerting anti-inflammatory effects. However, in the present study, the essential oil of fleagrass exhibited inhibitory activity against COX-2 enzyme activity (with an inhibition rate of 30% at a concentration of 50 μg/mL) and NO release (with an inhibition rate of 44% at a concentration of 50 μg/mL) (data not shown). These findings suggested that the essential oil of fleagrass possessed anti-inflammatory potential; however, the underlying mechanisms need further exploration. A few studies have been conducted on the non-volatile components of fleagrass. Ma [4] isolated and identified 46 compounds from methanol and petroleum ether extracts, which were mainly common natural products, such as lignans, phenolic acids, phenylpropanoids, and sterols. The present results showed that the aqueous, methanol, and ethyl acetate extracts of fleagrass, especially the ethyl acetate extract, exhibited significant anti-inflammatory activity. Hence, we plan to continue isolate and identify these extracts for further investigation.

Traditionally, the Akha people of Mengla intercrop fleagrass with upland rice in swidden fields. However, owing to social change factors in recent years such as the growing population and cash crop plantation and related new policies, this traditional swidden agriculture in China is gradually being replaced and/or disappearing [3]. Along with societal development, modern commodities are gradually replacing traditional materials. Therefore, the existence and use of fleagrass are under threat and are disappearing in the Akha villages of China before we paid attention to it. In addition to fleagrass, many other forms of traditional knowledge within local communities are facing the same challenges; hence, they must be documented, valued, verified, and developed. Although the importance of these precious traditional knowledge has declined in the daily lives of indigenous people because of various factors, its value is incredible. Ethnobotanical knowledge can provide irreplaceable clues for the discovery of useful products. Our studies on fleagrass provide a reference model for other studies on traditional knowledge preservation. Traditional knowledge can be preserved through ethnobotanical investigation, and the verification and exploration of traditional knowledge using modern techniques can enhance our understanding of these cultural heritages; the beneficial research outcomes obtained can revitalize the traditional knowledge, enhance ethnic cultural confidence and new development interests of the local community, and provide new and better choices for various human needs.

In conclusion, this study is the first to indicate that fleagrass exerts a remarkable anti-inflammatory effect by interfering with the conversion of AA and the activation of TLR4/MyD88/NF-κB signalling pathways. This experimental evidence provides a new option for treating inflammation, while also expanding the potential applications of fleagrass, such as inflammation being one of the causes of numerous skin issues, thereby our findings enhancing the therapeutic value of fleagrass in skin care, cosmetics, tissue regeneration, and various dermatological diseases. Concurrently, it presents a unique opportunity to safeguard and pass down this invaluable traditional knowledge to future generations.

## Materials and methods

### Chemicals and reagents

Arachidonate 5-lipoxygenase (5-LOX), horseradish peroxidase (HRP), and arachidonic acid (AA) were purchased from Sigma-Aldrich (St. Louis, MO, USA), Xue Man Biotechnology Development Co., Ltd. (Shanghai, China) and TCI Development Co., Ltd. (Shanghai, China), respectively. 3′3′5′5-tetramethylbenzidine (TMB) and nordihydroguaiaretic acid (NDGA) were purchased from Adamas-beta Reagent Co., Ltd (Shanghai, China) and Aladdin Biochemical Technology Co., Ltd. (Shanghai, China), respectively. The cyclooxygenase 2 (COX-2) inhibitor screening kit (S0168) and BCA Protein Assay Kit were purchased from Beyotime Biotechnology Co., Ltd. (Shanghai, China). Dulbecco’s modified Eagle medium (DMEM) containing 10% foetal bovine serum (FBS) 1% penicillin–streptomycin solution were and 1% GlutaMax was purchased from Procell Life Science and Technology Co., Ltd. (Wuhan, China). LPS (*Escherichia coli* 055: B5), dexamethasone, and dimethyl sulfoxide (DMSO) were purchased from Solarbio Science and Technology Co., Ltd. (Beijing, China). Cell Counting Kit-8 (CCK-8) was purchased from Biosharp (Anhui, China). Enzyme-linked immunosorbent assay (ELISA) kits (interleukin (IL)-6, IL-10, tumour necrosis factor (TNF)-α) were purchased from Multi Sciences Biotech Co., Ltd. (Hangzhou, China). Griess Reagent Kit for Nitrite Determination was purchased from Thermo Fisher Scientific, Inc. (NY, USA). Glyceraldehyde-3-phosphate dehydrogenase (GAPDH) and toll-like receptor (TLR4) antibodies were purchased from GenScript Biotech Corp. (Nanjing, China) and Proteintech Group, Inc. (Wuhan, China), respectively. Inducible nitric oxide synthase (iNOS) and MyD88 antibodies were purchased from Affinity Biosciences LTD. (Jiangsu, China). P65 and P-p65 antibodies were purchased from Abcam (Shanghai, China). carrageenan was purchased from Aladdin Biochemical Technology Co., Ltd. (Shanghai, China).

### Plant materials

Fleagrass was collected in the Menglun Town, Xishuangbanna Dai Autonomous Prefecture, Yunnan Province, China (21° 90′ N, 101° 27′ E) and identified by the prof. Pei Shengji of Kunming Institute of Botany (KIB), Chinese Academy of sciences (CAS) in October 2021. The plant specimens were stored at Herbarium of KIB-CAS (specimen number: 1341141).

### Preparation of the extract

The fresh fleagrass plants were divided into two parts: the inflorescence and the stem and leaves. The two parts of the plant material were cut separately, heated in distilled water for 3 h at 100 ℃ according to traditional usage methods. The filtered aqueous solution was concentrated to obtain the aqueous extracts of the inflorescence (ST-F) and those of the stem and leaf (ST-SL).

Dried inflorescence and stem-leaf powders were soaked in 95% methanol with ultrasonic wave for 30 min, respectively. The filtrate was concentrated and then added to the mixture of distilled water and petroleum ether (1:1). After full extraction is completed, the aqueous part of the extraction is obtained, and an equal amount of ethyl acetate solution is added to this aqueous part for further extraction. After extraction, the ethyl acetate layer was collected, which was evaporated and concentrated to form paste, and then the extract of the ethyl acetate parts of inflorescence (YSYZ-F) and stem-leaf (YSYZ-SL) was obtained respectively. The above residue of 95% methanol was soaked again in 65% methanol with ultrasonic wave for 30 min, respectively. Then the collected filtrate was concentrated into inflorescence 65% methanol extract (JC-F) and stems-leaves 65% methanol extract (JC-SL).

### 5-LOX analysis

All test samples (ST-F, ST-SL, YSYZ-F, YSYZ-SL, JC-F, and JC-SL) were dissolved in 100% DMSO and diluted with Tris–HCl to 250 μg/mL. The final concentration of DMSO should not exceed 5%. Initially, the extract solution, HRP, and 5-LOX (each 10 μL) were incubated with 160 μL of Tris–HCl buffer (0.1 M PH = 7.0) at 37 ℃ for 10 min. Then, TMB and AA (10 μL each) were added to the mixture and incubated at 37 ℃ for 15 min. Finally, the reaction was terminated by adding 10 μL of H_2_SO_4_. Their optical density (OD) values were determined at 450 nm using an enzymatic assay system. The rate of the inhibition of 5-LOX activity was calculated as follows:

$${\text{Inhibition rate}}\left( \% \right) = {1 - }\left( {{\text{OD}}_{{{\text{sample}}}} {\text{ - OD}}_{{\text{sample background}}} } \right)/\left( {{\text{OD}}_{{{\text{control}}}} {\text{ - OD}}_{{{\text{blank}}}} } \right) \times {1}00$$

### COX-2 analysis

The test samples were prepared in the same manner as in the 5-LOX experiment and diluted to 50 μg/mL with a buffer solution. The inhibition of COX-2 activity in the test samples was determined using the COX-2 Inhibitor Screening kit [44]. Briefly, the blank control, 100% enzyme activity control, and sample group were set up, and the specific experimental steps were performed according to the manufacturer’s instructions. The rate of the inhibition of COX-2 activity was calculated as follows:

$${\text{Inhibition rate}}\left( \% \right) = \left( {{\text{OD}}_{{{1}00\% {\text{ enzyme activity}}}} {\text{ - OD}}_{{{\text{sample}}}} } \right)/\left( {{\text{OD}}_{{{1}00\% {\text{ enzyme activity}}}} {\text{ - OD}}_{{\text{blank control}}} } \right) \times {1}00$$

### Cell culture of RAW 264.7

The mouse monocyte–macrophage cell line, RAW264.7, was purchased from Procell Life Science and Technology Co., Ltd. (Wuhan, China). The cells were cultured in DMEM supplemented with 10% FBS and 1% double antibody (100 U/mL penicillin and 100 μg/mL streptomycin) in a 5% CO_2_ incubator at a constant temperature of 37 °C. Stock solutions of the extracts were prepared at a concentration of 50 mg/mL in 100% DMSO. Working concentrations of the extracts were then diluted using the culture medium. The final DMSO concentration was 0.2%.

### Cell viability assays

The cytotoxic effects of the test samples (ST-F, ST-SL, YSYZ-F, YSYZ-SL, JC-F, and JC-SL) on RAW264.7 macrophage cells were investigated by performing the CCK-8 assay. The cells were randomly inoculated in 96-well plates (5 × 10^4^ cells/well) for 24 h. Afterwards, the cells were co-incubated with the test samples, DEX, and LPS (1 μg/mL) for 24 h. Further, the CCK-8 solution (10 µL) was added to each well and incubated for another 1.5 h according to the manufacturer’s instructions. Absorbance was measured at 450 nm using a microplate reader, and cell viability was calculated according to the following formula:

$${\text{Cell viability}}\left( \% \right) = \left( {{\text{OD}}_{{{\text{treatment}}}} {\text{ - OD}}_{{{\text{blank}}}} } \right)/\left( {{\text{OD}}_{{{\text{control}}}} {\text{ - OD}}_{{{\text{blank}}}} } \right) \times {1}00\% .$$

### Nitric oxide (NO) analysis

The concentration of NO in the culture supernatant was measured using the Griess reagent system kit according to the manufacturer’s instructions. Briefly, the cells were randomly inoculated into 24-well plates (2 × 10^5^ cells/well) for 24 h. After 2 h of treatment with the test samples (ST-F, ST-SL, YSYZ-F, YSYZ-SL, JC-F, and JC-SL) and DEX, LPS (1 μg/mL) was added to the RAW264.7 cells and cultured for 22 h. Supernatants were collected and measured according to the manufacturer’s instructions using a commercial kit.

### Measurement of the levels of IL-6, TNF-α, and IL-10 by ELISA

The cells were randomly inoculated into a 6-well plate at a cell density of 5 × 10^5^/well for 24 h, followed by incubation with different concentrations of YSYZ-F (4 and 12 μg/mL) and LPS (1 μg/mL) for 24 h. Supernatants were collected and used to measure the levels of IL-6, TNF-α, and IL-10 according to the manufacturer’s instructions.

### Western blotting

Cell treatment was the same as that used in the ELISA. Cell proteins were prepared in cold phosphate-buffered saline (PBS) and radioimmunoprecipitation assay buffer containing phenylmethanesulfonyl fluoride. The protein samples were separated using sodium dodecyl sulphate–polyacrylamide gel (12%) and transferred to a polyvinylidene difluoride membrane. Further, the TBST membrane containing 5% skim milk power for 2 h and incubated with the corresponding primary antibodies (iNOS, P65, P-p65, TLR4, MyD88, and GAPDH) at 4 ℃ for 24 h, followed by incubation with horseradish peroxidase-conjugated secondary antibodies for 2 h. The proteins were detected and colour development was observed using the ECL system.

### Immunofluorescence assay

Coverslips were placed on 6-well plates containing the test samples and cell treatment was the same as that used in the ELISA. The cells on the slides were soaked in PBS, fixed with 4% paraformaldehyde, soaked with 0.5% Triton X-100 for 20 min and then closed with normal goat serum for 30 min at room temperature (25 ℃). Subsequently, the coverslips were cleaned with PBS and incubated with the corresponding primary antibodies (nuclear factor-kappa B (NF-κB) p65)) for 24 h at 4 °C, followed by incubation with a cy3-labeled secondary antibody for 1 h. 4′,6-diamidino-2-phenylindole staining was performed and images were analyzed under a fluorescence microscope.

### Animals and treatment

Male Kunming mice (18–22 g) were obtained from the Experimental Animal Center of Kunming Medical University, Yunnan Province, China. Mice were exposed to specific pathogen free environment for 12 h dark/light cycle at a constant temperature and humidity. Mice are allowed free access to standard laboratory food and water. The animal experiments were approved by the Research Ethics Committee of the Kunming Institute of Botany (kib202303018), Chinese Academy of Sciences. The mice were acclimatised to their new environment for 1 week before the experiment.

### Carrageenan-induced mouse paw oedema model

An experimental method reported in previous studies was used with slight modifications [45, 46]. Briefly, the mice were randomly divided into the following five groups (n = 5): model group, YSYZ-F groups, and positive control (DEX). YSYZ-F dissolved in acetone was used for the treatment. 20 µL each of different concentrations (4 mg/mL, 8 mg/mL and 12mg/mL) YSYZ-F and dexamethasone (0.1 mg/paw) were applied to the right hind paw of mice, and the model groups were treated with the same amount of acetone. After 1 h, each mouse was injected with carrageenan (1% in saline) (50 μL) on the right hind paw. Basal thickness (T_0_) was measured before injection, whereas pathological thickness (T_t_) was measured 1, 2, 3, 4, 5, and 6 h after injection. The degree of swelling was calculated using the following formula:

$${\text{Paw oedema}}\left( \% \right) = \left[ {\left( {{\text{T}}_{{\text{t}}} {\text{ - T}}_{0} } \right)/{\text{T}}_{0} } \right] \times {1}00$$

### Statistical analyses

All data are expressed as the mean ± SEM of at least three independent experiments. One-way analysis of variance with Tukey’s test was used for multiple comparisons. Paw oedema data in Fig. 6 was analyzed with Two-way analysis of variance with Tukey’s test. All analyses were performed using GraphPad Prism 9.0 (Graphpad Software, Inc., USA). A *p*-value less than 0.05 was considered statistically significant.

## Data Availability

Data will be made available on request.

## Abbreviations

5-LOX: Arachidonate 5-lipoxygenase
COX-2: Cyclooxygenase 2
NO: Nitric oxide
IL: Interleukin
TNF: Tumour necrosis factor
ELISA: Enzyme-linked immunosorbent assay
NF-κB: Nuclear factor-kappa B
NSAIDs: Non-steroidal anti-inflammatory drugs
GC: Gas chromatography
LPS: Lipopolysaccharide
TMB: 3′3′5′5-Tetramethylbenzidine
NDGA: Nordihydroguaiaretic acid
DMEM: Dulbecco’s modified Eagle medium
FBS: Foetal bovine serum
DMSO: Dimethyl sulfoxide
PBS: Phosphate-buffered saline
IκB-α: Inhibitory protein factor kappa B alpha
HRP: Horseradish peroxidase
AA: Arachidonic acid
MED: Minimum effective dose
